# The Impact of Spinal Cord Nerve Roots and Denticulate Ligaments on Cerebrospinal Fluid Dynamics in the Cervical Spine

**DOI:** 10.1371/journal.pone.0091888

**Published:** 2014-04-07

**Authors:** Soroush Heidari Pahlavian, Theresia Yiallourou, R. Shane Tubbs, Alexander C. Bunck, Francis Loth, Mark Goodin, Mehrdad Raisee, Bryn A. Martin

**Affiliations:** 1 Center of Excellence in Design and Optimization of Energy Systems (CEDOES), School of Mechanical Engineering, College of Engineering, University of Tehran, Tehran, Iran; 2 Laboratory of Hemodynamics and Cardiovascular Technology, EPFL, Lausanne, Switzerland; 3 Children's of Alabama, Birmingham, Alabama, United States of America; 4 Department of Radiology, University Hospital of Cologne, Cologne, Germany; 5 Conquer Chiari Research Center, Dept. of Mech. Engineering, University of Akron, Akron, Ohio, United States of America; 6 SimuTech Group, Hudson, Ohio, United States of America; 7 Hydraulic Machinery Research Institute, School of Mechanical Engineering, College of Engineering, University of Tehran, Tehran, Iran; University of Antwerp, Belgium

## Abstract

Cerebrospinal fluid (CSF) dynamics in the spinal subarachnoid space (SSS) have been thought to play an important pathophysiological role in syringomyelia, Chiari I malformation (CM), and a role in intrathecal drug delivery. Yet, the impact that fine anatomical structures, including nerve roots and denticulate ligaments (NRDL), have on SSS CSF dynamics is not clear. In the present study we assessed the impact of NRDL on CSF dynamics in the cervical SSS. The 3D geometry of the cervical SSS was reconstructed based on manual segmentation of MRI images of a healthy volunteer and a patient with CM. Idealized NRDL were designed and added to each of the geometries based on *in vivo* measurments in the literature and confirmation by a neuroanatomist. CFD simulations were performed for the healthy and patient case with and without NRDL included. Our results showed that the NRDL had an important impact on CSF dynamics in terms of velocity field and flow patterns. However, pressure distribution was not altered greatly although the NRDL cases required a larger pressure gradient to maintain the same flow. Also, the NRDL did not alter CSF dynamics to a great degree in the SSS from the foramen magnum to the C1 level for the healthy subject and CM patient with mild tonsillar herniation (∼6 mm). Overall, the NRDL increased fluid mixing phenomena and resulted in a more complex flow field. Comparison of the streamlines of CSF flow revealed that the presence of NRDL lead to the formation of vortical structures and remarkably increased the local mixing of the CSF throughout the SSS.

## Introduction

99 years ago, neurosurgeon Harvey Cushing wrote in his seminal manuscript on cerebrospinal fluid (CSF) studies, “Our knowledge of the meningeal and ependymal coverings of the central nervous system, as well as of the part played by the fluid which circulates through and over them, has hardly kept pace with our knowledge of the nervous tissues which they envelop.” To this day, the complete physiological importance of the CSF dynamics remains enigmatic. A deeper knowledge of these dynamics could help to understand many pathologies such as Chiari malformation (CM) [1], [2], syringomyelia [3], [4], hydrocephalus [5], [6], spinal stenosis [7], Alzheimer's disease and multiple sclerosis [8] as well as intrathecal drug transport and delivery [9], [10]. At a more fundamental level, CSF dynamics have been thought to play a role in development [11], [12] of the brain and intracellular signaling associated morphogenesis of neural tissue [13].

CSF dynamics have been studied in the spinal subarachnoid space (SSS) to understand their pathophysiological importance in craniospinal disorders such as CM. Researchers have analyzed SSS CSF velocities *in vivo* [1], [14], [15], *in vitro* [16]–[18] and utilizing computational fluid dynamics (CFD) simulations *in silico* [19]–[22]. These efforts have given valuable information about the CSF movement with the long-term goal of determining objective and quantitative measures of disease severity.

Regarding *in vivo* studies in the SSS, various CSF flow features, such as inhomogeneous CSF flow (anterior dominance of the flow), synchronous bi-directional flow, and flow jets have been observed by 2D [14], [15], [23] and time-resolved three directional velocity-encoded phase contrast MR imaging (4D PC MRI) [1]. Nevertheless, due to the spatial resolution limit of the current MRI techniques, *in vivo* measurements have provided limited information on the impact of relatively small anatomical structures inside the SSS such as nerve roots and denticulate ligaments (NRDL). The anatomical size of these structures is too small to quantify at the present state-of-the art MRI resolution ∼0.5 mm isotropic resolution at 3T.

It is not clear what level of anatomical detail, e.g. 3D realistic NRDL geometry, is required to accurately reproduce *in vivo* CSF dynamics using CFD. However, many studies have employed CFD as a non-invasive tool to better understand the CSF dynamics in healthy and patient cases and statically analyze flow differences within each group. CFD has been applied with geometrically simplified [19]–[21] as well as subject-specific 3D anatomic models of SSS [10], [22], [24], [25]. These models have incorporated varying degrees of anatomical complexity. The study by Stockman [26] appears to be the only CSF flow CFD simulation in which idealized NRDL and arachnoid trabeculae were included in various configurations within an idealized concentric tube representing the SSS. At present, no studies in the literature have included anatomically realistic NRDL.

This study aims to determine the impact of NRDL on CSF dynamics using anatomically realistic 3D CFD models of the cervical spine. NRDL were modeled within a healthy subject and CM patient to understand the importance of these structures on CSF dynamics under a variety of hydrodynamic/disease states. Note, this study was not intended to analyze flow field differences between the CM patient and control. Rather, the aim was to investigate the impact of NRDL on CSF dynamics by comparing the CFD results with and without the NRDL included within each individual subject.

## Materials and Methods

The CFD model was based on subject specific MRI measurements of flow and geometry in the cervical spine obtained for a healthy subject and a CM patient. The healthy subject and patient geometries of this study were the same as that previously published by Yiallourou et al., in which these geometries were referred to as HVb and CM4, respectively [24]. However, in this study, idealized spinal cord NRDL were added to these geometries based on anatomical information obtained in the literature. CFD studies were conducted on the HVb and CM4 with and without the spinal cord NRDL representing four CFD geometries in total. The results for each simulation were compared with respect to the flow fields and several hydrodynamic parameters.

### Ethical statement

The MR data acquisition was performed at the Department of Radiology of Münster. The study was approved by the institutional review board of the University of Münster. Before the MR exams, written informed consent was obtained from the healthy volunteer and CM patient. Prior to further data processing MR data were anonymized.

### MRI CSF flow measurements

In order to obtain the flow boundary conditions for the CFD simulations, 4D PC MRI measurements were acquired in the cervical spine (from the foramen magnum (FM) to C7 vertebrae level) of a healthy male volunteer (22-years-old) with no history of neurological disorder or spinal trauma and a male clinically diagnosed CM patient (5-years-old) with a tonsillar herniation of 5.8 mm and symptoms corresponding to CM. The 4D PC MRI measurements were taken on a 1.5 T MRI scanner (Achieva 2.6 scanner, Philips, Best the Netherlands) using the sequence protocol developed by (Bunck et al. [1]). The subjects were asked to lie in the supine position in the scanner bed with a standard 16-channel head and neck coil. Flow velocities were encoded in anterior-posterior, in feet-head and in right-left direction. The 4D PC flow sequence acquired in both HVb and CM4 was aligned sagittally with the 3D stack covering the craniocervical junction and the entire cervical thecal sac. Scanning time ranged from 8 to 14 min depending on the heart rate of each subject and the velocity encoding gradient flow factor (VENC). CSF flow was quantified at nine axial locations along the spine for each subject. The axial location with the maximum peak flow was chosen as the inlet flow waveform boundary condition for the CFD simulation, based on the methods of Yiallourou et al. [24].

### MRI geometry measurements

A T2-weighted 3D, turbo spin-echo sequence (VISTA) with an isotropic spatial resolution of 0.8 mm defined the cervical spine geometry. Details of the MRI geometry measurement methods are included in Yiallourou et al [24].

### Subject-specific model of the dura and spinal cord

The three-dimensional anatomy of cervical SSS, containing the dura mater layer and spinal cord, was reconstructed from the T2-weighted MRI images by manual segmentation [24] using ITK Snap (version 2.2, University of Pennsylvania, Philadelphia, Pennsylvania, USA). The segmentation volume was extended ∼5 cm caudal to the C7 level to reduce entrance length effects. The resulting 3D surface model was smoothed by a Laplacian smoothing algorithm using MeshLab (version 1.3.2, Italy, Rome).

### Idealized NRDL model

The MRI resolution was insufficient to detect the NRDL. Thus, an idealized 3D model of the NRDL was created at each level along the spine using Autodesk Maya (version 2012, Autodesk Inc. California, USA) based on measurements reported in the literature [27], [28]. A summary of the spinal cord nerve root measurements is presented in Table 1. The sagittal MRI images were used to approximately identify the location of nerve roots along the spinal cord. Each dorsal nerve root consists of one row of rootlets with approximately 6–8 distinguishable rootlets in each nerve bundle [29]. A series of ridge-like features were added to the surface of dorsal nerve roots to mimic the presence of individual rootlets. The ventral nerve roots had two to three rows of rootlets with a large number of densely packed fine rootlets in each row [29]. This made it impractical to consider the effect of individual rootlets on the ventral nerve roots. Thus, the ventral nerve root surfaces were considered to be smooth.

**Table 1:** Summary of spinal cord nerve root measurements utilized in the 3D model of the cervical spine based on cadaveric measurements in the literature.

Spine Level	Location	Dorsal			Ventral		
		Radicular line length – RL (mm)	Median descending angle – θ (degrees)	Thickness (mm)	Radicular line length – RL (mm)	Median descending angle θ – (degrees)	Thickness (mm)
**C1**	*Left*	3.8	−0.9	0.65	8.9	−0.9	1.7
	*Right*	4.7	−1.8		7.9	−1.8	1.6
**C2**	*Left*	8.0	−17.1	0.70	12.3	−17.1	1.6
	*Right*	7.4	−18.2		12.6	−18.2	1.8
**C3**	*Left*	12.1	−43.4	0.73	11.6	−43.4	1.7
	*Right*	11.2	−39.4		12.1	−39.4	1.7
**C4**	*Left*	12.3	−44.4	0.75	12.1	−44.4	1.7
	*Right*	12.7	−38.6		12.3	−38.6	1.8
**C5**	*Left*	12.5	−42.9	0.81	13.1	−42.9	2.0
	*Right*	12.1	−37.7		13.4	−37.7	1.9
**C6**	*Left*	11.8	−44.5	0.90	13.5	−44.5	1.9
	*Right*	12.3	−41.5		13.5	−41.5	1.9
**C7**	*Left*	11.6	−51.6	0.98	12.0	−51.6	1.9
	*Right*	11.4	−47.6		12.4	−47.6	2.0
**C8**	*Left*	11.2	−58.5	0.89	11.1	−58.5	1.9
	*Right*	10.8	−55.9		11.1	−55.9	1.9

Detailed measurements of denticulate ligament geometry were not available in the literature. Hence, their dimensions and location along the spine were approximated based on anatomical photos [30], [31]. Denticulate ligaments were modeled as thin membranes extending from spinal cord to the dura in the space between nerve roots at each cervical level with a slight concavity toward the posterior side of the spinal cord [32]. The thickness of these membranes was considered as the smallest value allowed by geometrical constraints (approximately 0.1–0.15 mm).

### Composite model

**Figure 1:**
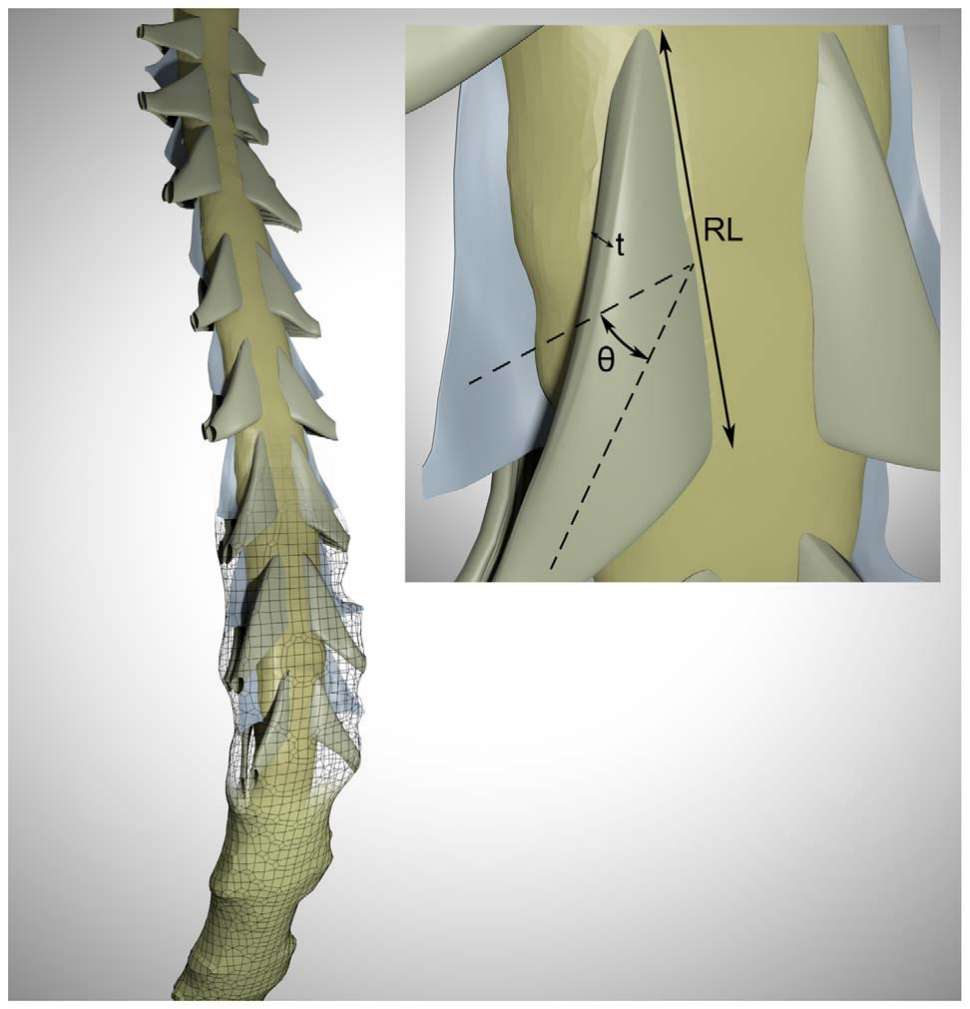
Rendering of the 3D geometry of cervical SSS in the healthy subject containing idealized nerve roots and fine structures. The dimensions of these structures are based on anatomical measurements in Table 1. Meshwork delineates the dural surface and the top portion is transparent to better visualize the anatomy. Inset represents the extracted dimension from the medical literature (θ = median descending angle, t = nerve root thickness, RL = radicular line length).

The NRDL model and subject-specific model of the spinal cord and dura were combined to form a composite model representing the cervical SSS for the CM patient and healthy subject with fine structures (Figure 1). Scaling was required to place the NRDL within each model. In order to implement the NRDL model into the patient (younger) case, they were scaled based on the relative sizes of the overall geometry in each cervical level (with scaling coefficient ranging from 0.70 to 0.85). Additionally, the NRDL orientation at each level along the spine was fine-tuned to be located at the spinal cord mid-line in the dorsal-ventral direction.

### CFD simulations

The model geometries were imported into ANSYS ICEM CFD (version 14.0, ANSYS Inc., Canonsburg, USA) and a non-uniform unstructured computational mesh consisting of 7.4 and 5.5 million tetrahedral elements was generated for the healthy and Chiari model, respectively. Careful attention was given to use a higher resolution mesh in regions with higher spatial complexity such that the mesh element size varied from approximately 0.1 mm near the walls to 0.5 mm in the central region of the SSS.

For CFD simulations, a commercial finite volume solver ANSYS Fluent (version 14.0, ANSYS Inc., Canonsburg, USA) was used. CSF flow was assumed to be laminar with an incompressible Newtonian fluid of the same properties as water at body temperature [33], [34]. The flow is expected to be laminar since the maximum Reynolds number based on internal flow in a pipe, 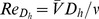, was calculated to be 187 and 352 for the healthy and patient cases, respectively. In this equation, 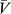 is the temporal maximum of the spatial mean velocity at a given axial plane along the spine computed from CSF flow boundary condition and cross-sectional area, *ν* is the kinematic viscosity, and 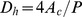 is the hydraulic diameter with cross sectional area, 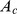, and wetted perimeter, *P* determined from the SSS geometry.

A pressure-based solver was implemented with second order of accuracy scheme in space and time. SIMPLE method was used for velocity-pressure coupling along with Green-Gauss Node based method to evaluate the gradient terms in the Navier-Stokes equations. The time-step size was chosen to be *T*/100, where *T* represents the length of one CSF flow cycle. No slip (zero velocity) boundary conditions (BC) were specified at the walls. A zero pressure BC was assumed at the flow outlet at the cranial end of the geometry because it was not possible to obtain pressure non-invasively *in vivo*. Thus, we only quantified the pressure gradients within each model. A time-dependent velocity inlet BC based on the subject-specific MRI flow measurements was specified at the caudal end of each model. The flow initial conditions were chosen to be zero pressure and velocity throughout the domain. The scaled residual was set to 10^−6^ as a convergence criterion for the iterations in each time-step. Results are presented based on the third flow cycle to minimize startup effects.

### Hydrodynamic parameter assessment

**Figure 2:**
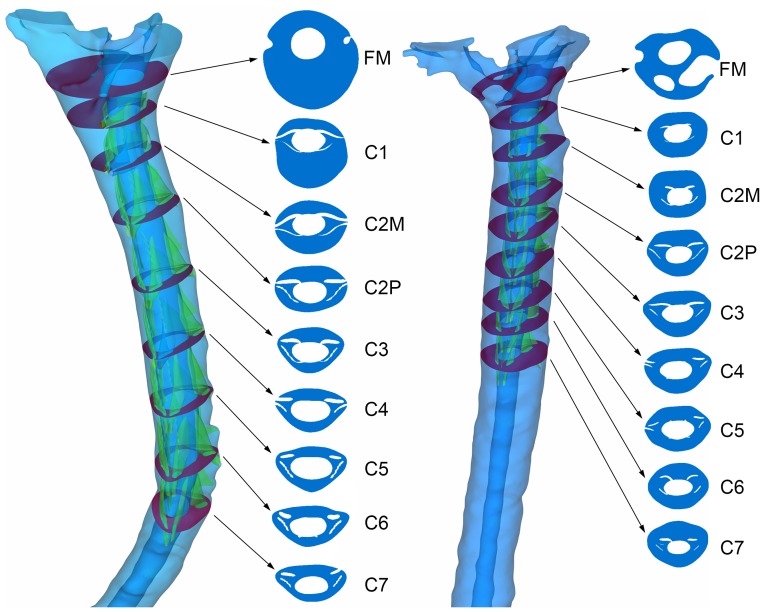
3D geometry of the cervical SSS and the selected axial planes (violet) for the healthy (left) and patient (right) case. Spinal cord NRDL are shown in green (FM = foramen magnum, C2M = middle of 2^nd^ cervical vertebra, C2P = junction of C2/C3 vertebra).

We assessed the impact of the presence of spinal cord NRDL on the velocity profiles in the axial, coronal and sagittal planes for the healthy and CM model. In addition, the following hydrodynamic parameters for all CFD simulations were quantified:

- **Dimensions.** The hydraulic diameter, 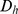, and cross-sectional area, 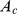, were calculated at nine axial planes located along the spine (FM, C1, C2M, C2P, C3, C4, C5, C6, C7), as shown in Figure 2.
- **Peak systolic and peak diastolic velocity.** The maximum velocity magnitude within an axial cross-section (FM to C7) at two time points, peak CSF flow in the caudal (systole, 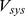) and cranial (diastole, 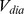) directions, were determined.
- **Bidirectional velocity.** At a given axial plane, the maximum instantaneous difference between caudal versus cranial directed CSF velocity, 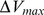 is defined as the magnitude of bidirectional velocity. The duration of bidirectional was quantified as the time span for which the magnitude of bidirectional velocity exceeded 1.0 mm/s for each plane.
- **Local Reynolds number for internal flow.** As described above, Reynolds number based on hydraulic diameter was quantified at each axial plane (FM to C7).
- **Local Reynolds number for external flow**. For the CFD simulations with NRDL, a local Reynolds number based on external flow over a surface was computed at each of the dorsal and ventral nerve roots as well as the denticulate ligaments (e.g. 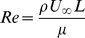). Characteristic length, *L*, was defined by the length of the flat surface of the nerve root or ligament midway between the spinal cord and dura. The free stream velocity, 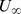, was defined as the velocity just upstream at peak systolic CSF flow.
- **Secondary flow parameter.** To help compare the level of fluid mixing with and without NRDL, a secondary flow parameter was defined as: 
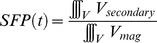
, where 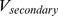 and 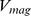 are 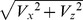 and 
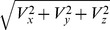
, respectively; note the *y*-direction is axial and approximately the streamwise direction. The volume integral was obtained over the SSS volume between each axial plane (FM-C1, C1-C2M, C2M-C2P, C2P-C3, C3-C4, C4-C5, C5-C6, C6-C7).
- **Peak wall shear stress.** Based on the gradients of all three components of velocity normal to the wall, the peak magnitude of wall shear stress, 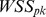, was determined between each axial plane. 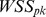 was obtained utilizing the EnSight (version 10.0, CEI Inc., Apex, USA) built-in functions for calculating velocity gradients and geometrical wall normal direction.
- **Pressure gradient.** Longitudinal pressure dissociation, or pressure gradient, has been the subject of previous *in vivo*, *in vitro* and *in silico* studies of spinal CSF dynamics [16], [19], [35]–[41]. We obtained the unsteady pressure gradient across the model from the FM to C7 level, Δ*P*(*t*). We also quantified the peak pressure gradient over the cardiac cycle that occurred between each axial plane.
- **Integrated longitudinal impedance (ILI).** Longitudinal impedance, *Z_L_*, is a representation of resistance to pulsatile flow. To obtain *Z_L_*, we performed a fast Fourier transform (FFT) on the input flow waveform, *Q*(*t*), and the pressure gradient for each axial segment, Δ*P*(*t*), resulting from the CFD calculation where, 
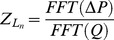
. The FFT coefficients of Δ*P_n_* were divided by the flow waveform FFT coefficients, *Q_n_*, in complex form for each harmonic separately. *Z_L_* was determined based on the summation of the longitudinal impedance moduli, 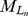, for each harmonic n = 1 to 8 Hz, where 
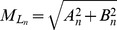
. This methodology is based on the vein graft patency studies by Meyerson et al. [42] and Skelly et al. [43] and utilized in the thesis work of Kalata [44].
- **Vortex Shedding Frequency.** The unsteady velocity, *V*(*t*), caudal to the NRDL from C1 to C8 was examined to determine if vortex shedding was present in the flow field.

### Independence studies

**Figure 3:**
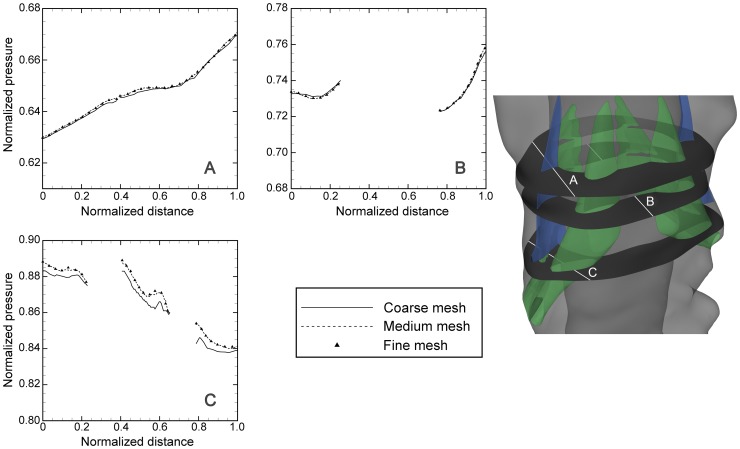
Plots of normalized pressure along paths through SSS (showed in the right inset) as calculated with different grid refinements (Table 2) at peak systole. Green and blue areas denote nerve roots and denticulate ligaments, respectively.

Grid, time-step and cyclic independence studies were carried out using the following methods. To evaluate grid independence, three different mesh resolutions, 0.53, 1.8 and 3.5 million cells, were used on a truncated geometry of the full model that included one pair of denticulate ligaments and nerve roots (∼7 cm segment extended caudally and cranially over C6 nerve roots, Figure 3). Pressure and velocity contours at three cross-sections of the flow domain were compared at different points during the cardiac cycle at selected paths (Figure 3). Pressure values are normalized by the peak value of their corresponding cross section.

Time-step independence was assessed by carrying out the computations for the second period using time-step sizes of T/50, T/100 and T/200 where T is the length of one cardiac cycle. The time-step size of T/100 proved to be sufficient and was used for the subsequent studies.

The results obtained in the first three periods were compared in order to evaluate period independence. The results of independence studies are presented in Table 2. The relative error, *e*, in Table 2 is defined as [45]:
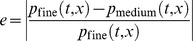
Where *p* is the pressure calculated at spatial position *x* and time within the cardiac cycle *t*. The subscripts “medium and “fine” refer to calculations carried out with the medium and fine grid respectively. Table 2 also indicates the corresponding time-step size and number of periods for other independence studies.

**Table 2:** Summary of independence study simulation parameters.

Independence study	Parameter to study	Constant parameter	Relative error estimate (%)
	0.53M		
Mesh size (MS): number of elements in truncated geometry	1.8M	PN = 2, Δt = T/100	<3.0
	3.5M		
	1		
Period number (PN)	2	MS = 1.8M, Δt = T/100	<1.5
	3		
	T/50		
Time-step size (Δt) (s)	T/100	MS = 1.8M, PN = 2	<2.0
	T/200		

(MS = mesh size, PN = period number, M = million cells, T = cycle period in seconds, Δt = time-step size in seconds).

## Results

**Figure 4:**
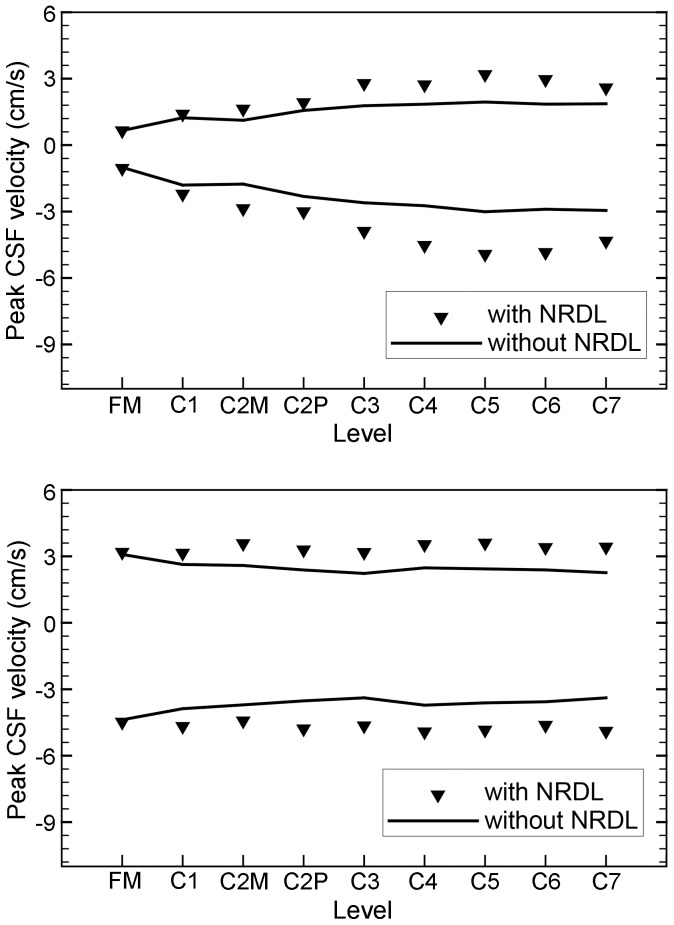
Peak systolic and diastolic CSF velocity for the Healthy (top) and Chiari I malformation patient along the spine with and without NRDL.

**Figure 5:**
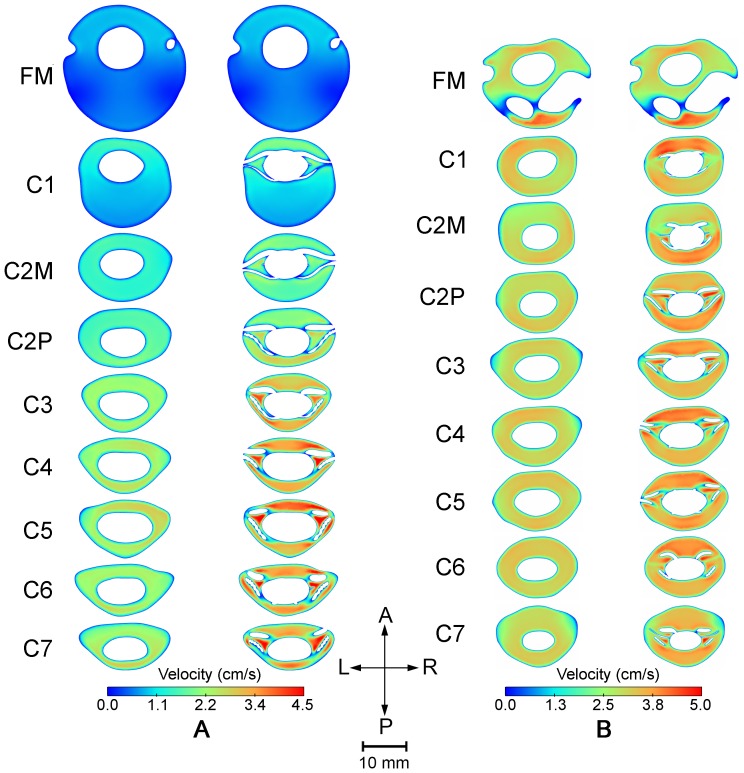
Velocity magnitude contours at different locations along cervical SSS plotted at the time corresponding to the peak systole for the healthy case (A) and patient diagnosed with Chiari I malformation (B). In each set of contours, the left and right column represent the results for cases without and with NRDL, respectively.

**Figure 6:**
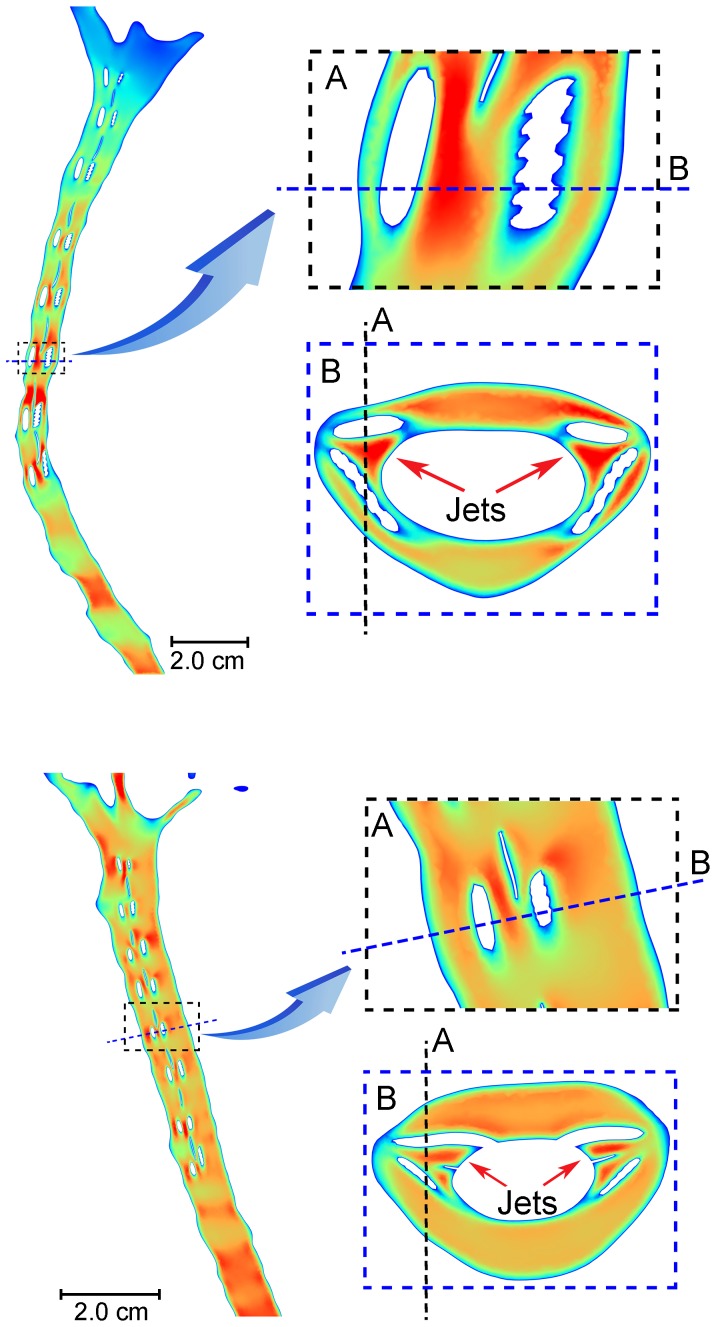
Sagittal view for the healthy (top) and patient (bottom) subject showing the location of flow jets between dorsal and ventral nerve roots.

**Figure 7:**
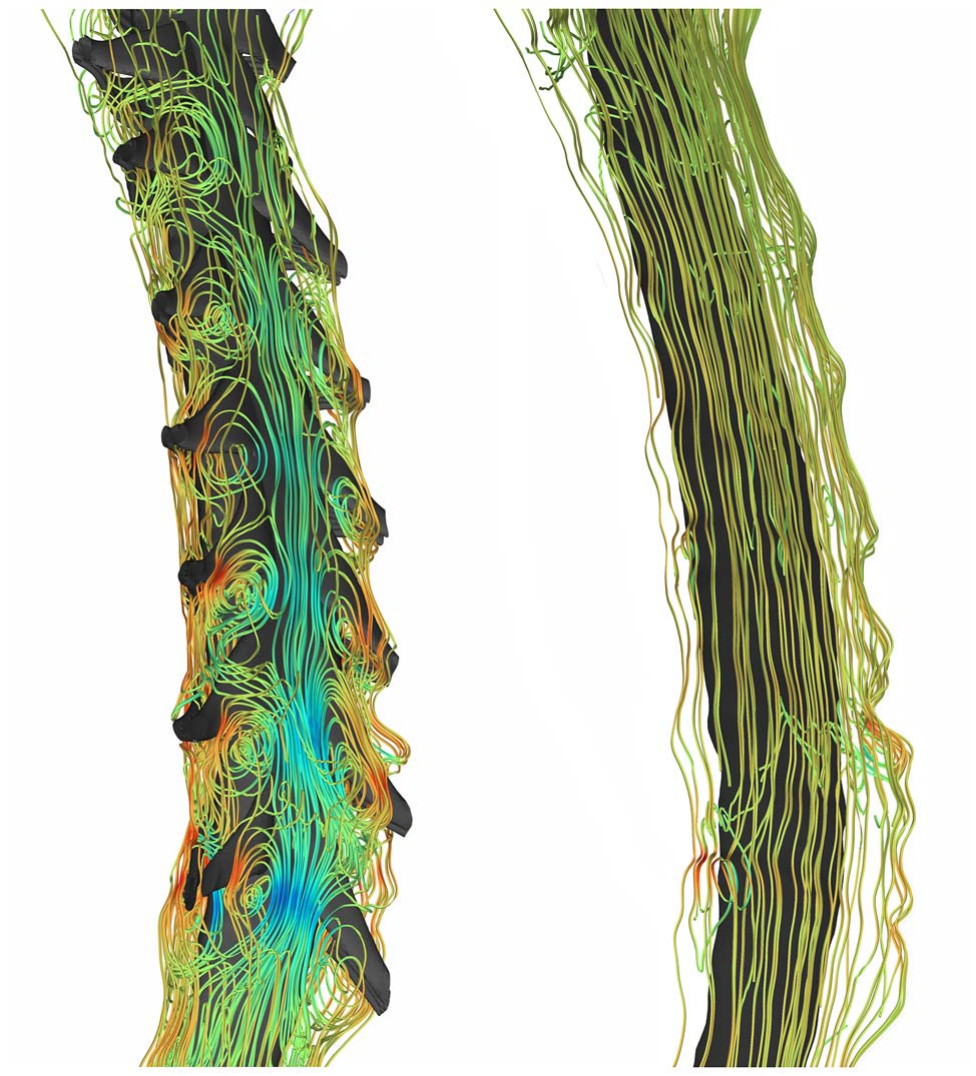
Streamline plots for healthy case with fine structures (left) and without fine structures (right). Streamlines are calculated at t = 0.56 s.

Overall, the addition of NRDL decreased 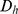 and 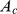 and this change was greatest moving caudally away from the FM (Table 3, Table 4). Similar to 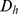 and 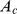, the difference in the values of 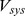 was present below the C1 level; the location where the first nerve root was placed. Both 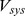 and 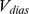 increase with the addition of NRDL, with an average 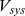 increase of 30 and 21% in healthy and patient case, respectively and an average 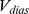 increase of 39 and 36% in the healthy and patient case, respectively. The impact of NRDL on the peak systolic and diastolic velocities at different levels is shown in Figure 4. Velocity magnitudes at peak systolic CSF flow show a difference with and without NRDL at axial cross-sections caudal to FM (Figure 5) for both healthy and patient cases. Velocity profiles for both cases in a sagittal view reveal the complex flow patterns present with NRDL (Figure 6). The cases with NRDL had greater velocities on the anterior side of the spinal cord and local increases in velocity between dorsal and ventral nerve roots. The presence of NRDL dramatically alters the CSF flow streamlines and has a mixing/stirring effect on the flow field as CSF moves up and down in the spinal canal (Figure 7).

**Table 3:** Healthy case hydrodynamic parameters with (W) and without (W/O) NRDL at each axial level (left) and within each spine segment (right).

Axial level	D_h_ (mm)		A_c_ (mm^3^)		V_sys_ (cm/s)		Spine segment	SFP		ΔP_peak_ (Pa)		ILI (dyne/cm^5^)	
	W	W/O	W	W/O	W	W/O		W	W/O	W	W/O	W	W/O
**FM**	17.8	17.8	610	610	1.0	1.0	***FM-C1***	0.39	0.35	1.2	1.2	40	41
**C1**	8.0	13.4	313	333	2.2	1.8	***C1-C2M***	0.42	0.43	2.7	2.4	87	83
**C2M**	5.3	10.5	206	238	2.9	1.8	***C2M-C2P***	0.46	0.45	4.3	3.7	137	127
**C2P**	4.5	9.2	175	203	3.0	2.3	***C2P-C3***	0.40	0.31	6.8	6.0	217	204
**C3**	3.7	7.5	134	157	3.9	2.6	***C3-C4***	0.35	0.24	7.9	6.6	251	225
**C4**	4.0	6.8	129	150	4.5	2.7	***C4-C5***	0.32	0.22	7.2	5.9	226	202
**C5**	3.7	6.5	128	145	4.9	3.0	***C5-C6***	0.34	0.22	9.2	7.1	288	242
**C6**	3.9	7.2	138	159	4.8	2.9	***C6-C7***	0.44	0.28	8.1	6.2	252	209
**C7**	4.6	7.6	143	158	4.3	2.9							

(D_h_ = hydraulic diameter, A_c_ = cross sectional area, V_sys_ = peak systolic velocity, SFP = secondary flow parameter – defined in text, ΔP_peak_ = peak pressure gradient, ILI = integrated longitudinal impedance).

**Table 4:** Chiari I malformation case hydrodynamic parameters with and without NRDL at each axial level (left) and within each spine segment (right).

Axial level	D_h_ (mm)		A_c_ (mm^3^)		V_sys_ (cm/s)		Spine Segment	SFP		ΔP_peak_ (Pa)		ILI (dyne/cm^5^)	
	W	W/O	W	W/O	W	W/O		W	W/O	W	W/O	W	W/O
**FM**	7.2	7.2	267	267	4.5	4.4	***FM-C1***	0.50	0.47	4.0	4.1	83	88
**C1**	7.2	9.1	176	180	4.7	3.9	***C1-C2M***	0.28	0.16	4.4	4.1	90	90
**C2M**	6.7	9.8	184	193	4.4	3.7	***C2M-C2P***	0.29	0.24	5.7	5.2	118	113
**C2P**	5.3	9.6	174	188	4.8	3.5	***C2P-C3***	0.33	0.30	4.5	4.2	93	92
**C3**	5.5	9.3	180	196	4.7	3.4	***C3-C4***	0.33	0.29	5.2	4.7	107	101
**C4**	6.2	9.0	178	187	4.9	3.7	***C4-C5***	0.34	0.29	5.0	4.6	102	101
**C5**	6.0	8.8	174	183	4.8	3.6	***C5-C6***	0.34	0.27	3.5	3.2	73	70
**C6**	6.2	9.3	170	179	4.6	3.6	***C6-C7***	0.35	0.28	4.8	4.4	100	96
**C7**	5.5	10.5	184	194	4.9	3.4							

The inclusion of fine structures increased the bidirectional velocity, both in the healthy volunteer and patient case. The average value of bidirectional velocity over all axial planes increased from 3.7±1.2 to 5.6±2.2 cm/s (mean ± SD) for the healthy, and from 5.8±0.4 cm/s to 7.6±0.8 cm/s for the patient case. Moreover, with NRDL included, the duration of bidirectional velocity was nearly identical for all planes and increased ∼0.09 to 0.21 s (130%) and 0.07 to 0.11 s (60%) for the healthy and the patient, respectively.

**Figure 8:**
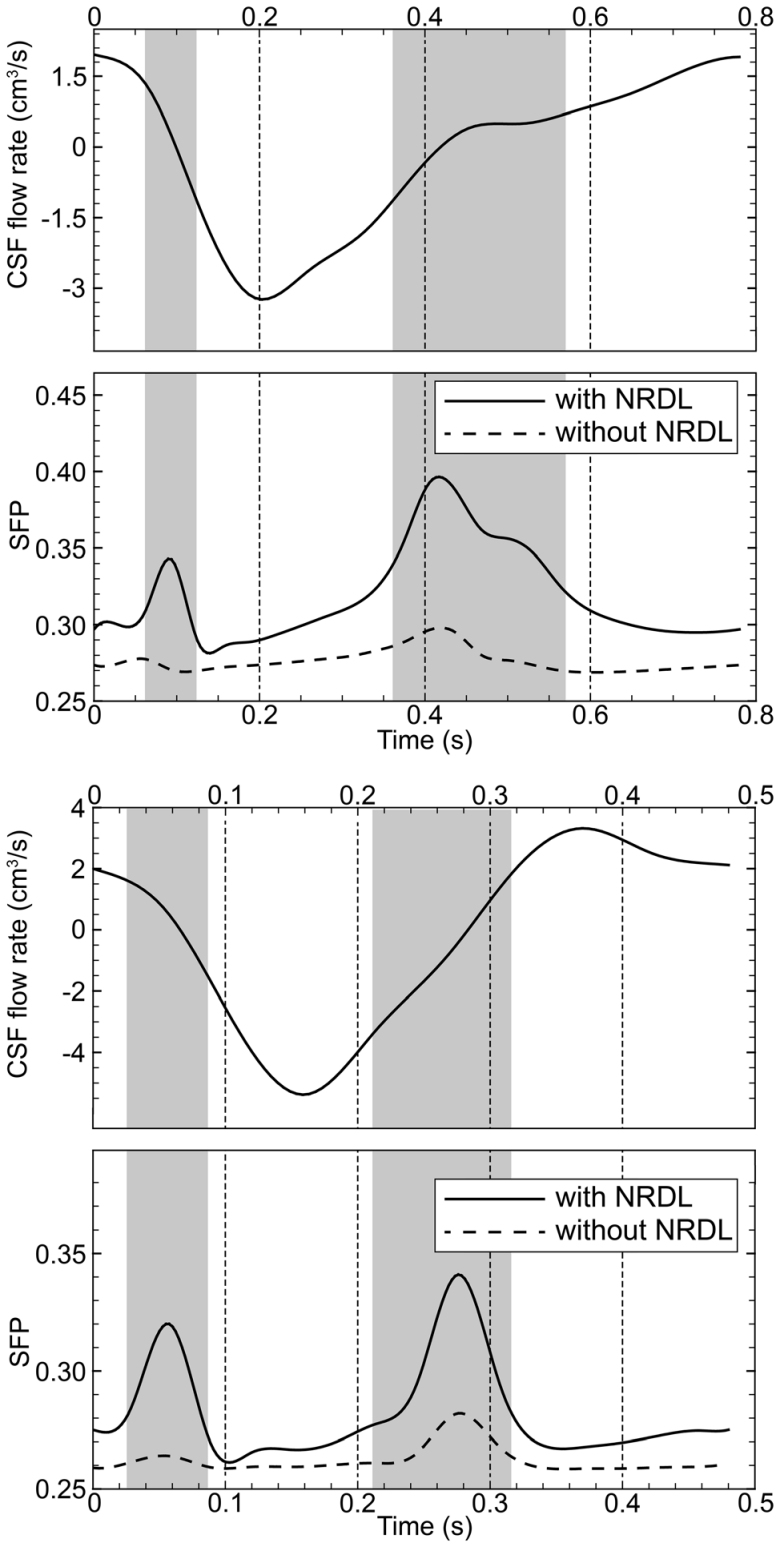
Secondary flow parameter (SFP) during cardiac cycle with and without NRDL for the healthy (top) and patient cases (bottom). Gray area highlights the duration of bidirectional flow in the presence of the NRDL for each case. Note that SFP is largest due to the change in flow direction.

Peak values of SFP along the cervical SSS are reported in Table 3 and Table 4 for the healthy and patient cases, respectively. The average value of SFP increases 21 and 18% in healthy and patient cases respectively with NRDL present. SFP calculated in the region between FM and C7 over the cardiac cycle showed even greater difference with and without NRDL (Figure 8) in both healthy and patient cases. The SFP value was elevated in the presence of the NRDL with an average increase of 15.1 and 7.1% in healthy and patient cases, respectively. Note that the impact of NRDL on SFP is greatest when the CSF flow changes direction.

Peak wall shear stress (WSS) averaged throughout different segments of cervical SSS increased from 0.22±0.12 and 0.30±0.03 to 0.52±0.23 and 0.87±0.19 Pa for healthy and patient cases, respectively. Peak WSS primarily occurred on the surface of NRDL and on the pia mater surface in the region between dorsal and ventral nerve roots as a result of the flow jets (Figure 6). The maximum value of peak WSS throughout the cervical region increased from 0.49 to 0.89 Pa and 0.34 to 1.1 Pa for the healthy and patient cases, respectively with NRDL present.

**Figure 9:**
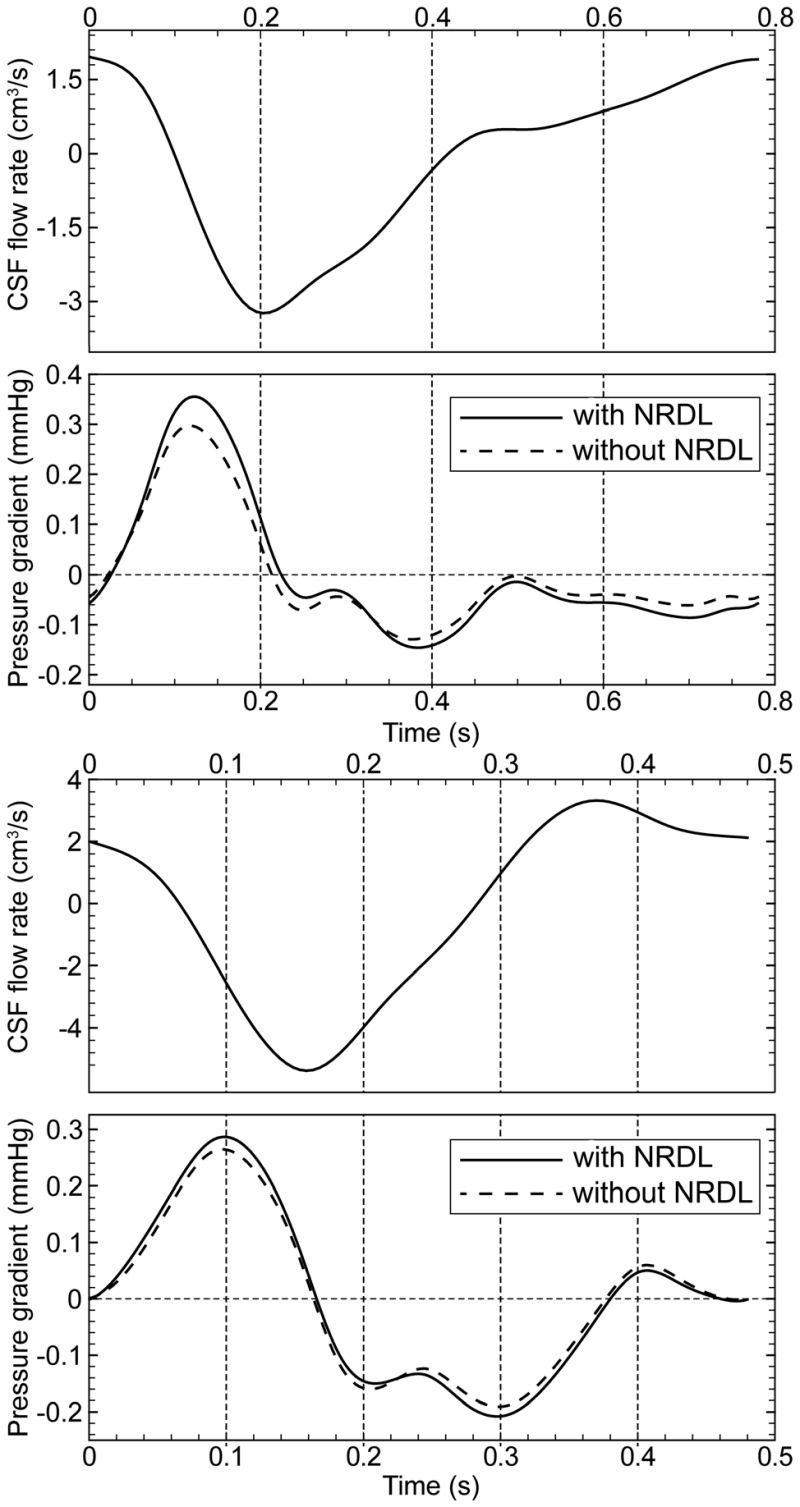
Unsteady pressure gradient comparison with and without NRDL in the healthy (top) and patient (bottom) subject.

The values of peak pressure gradient over the cardiac cycle (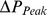) are reported in Table 3 and Table 4. Fine structures altered 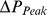 in both the healthy and patient cases with an average increase of 14.7 and 7%, respectively. The impact of NRDL on the pressure gradient along the cervical SSS, defined as the difference of mean pressure on FM and C7 planes, is shown in Figure 9. It was observed that the NRDL increased pressure gradient by an average of 18.4 and 8.5% for the healthy and patient case, respectively.

ILI increased with the addition of NRDL as shown for different segments of the cervical SSS (Table 3 and Table 4). The change of ILI in the healthy case was more evident compared to the patient case, specifically at segments further away from the FM. This was likely due to a smaller SSS cross-sectional area in the healthy case compared to the patient, particularly in lower levels of the spine where the addition of NRDL blocked a larger proportion of the area. Adding NRDL reduced the averaged cross-sectional area of the healthy and patient cases 12.1 and 5.4%, respectively (Table 3 and Table 4).

Note that the Mean velocity fluctuation frequency, assessed at the nine axial planes along the cervical spine caudal to the nerve roots (dorsal and ventral), was near the heart rate frequency at a value of 1.03±0.21 and 2.36±0.50 Hz for the healthy and patient case, respectively. Caudal to denticulate ligaments frequencies perturbations were measured to be 0.96±0.30 and 1.71±0.30 Hz for the healthy and patient case, respectively.

Mean Reynolds number for internal flow assessed along the cervical spine at the nine-axial planes (Figure 2) was 112±11 and 169±17 in the healthy subject with and without NRDL, respectively. Mean Reynolds number for internal flow was greater in the CM patient at 224±24 and 315±40 with and without NRDL, respectively. In the healthy subject, mean Reynolds number for external flow over the denticulate ligaments, dorsal nerve roots and ventral nerve roots was 243±100, 178±76 and 224±74, respectively. In the CM patient, mean Reynolds number for external flow over the denticulate ligaments, dorsal nerve roots and ventral nerve roots was 181±28, 150±35 and 173±28, respectively.

## Discussion

The presence of spinal cord nerve roots and denticulate ligaments (NRDL) has an important impact on CSF dynamics in the cervical spine. CFD simulations, under varying levels of anatomical complexity, have been utilized as a non-invasive method to evaluate CSF dynamics. However, these simulations require a great deal of anatomical simplification to perform. One aspect of the anatomy that often has not been considered in CFD simulations in the literature is NRDL. In the present study, we investigated the impact of NRDL, with 3D geometry, on CSF dynamics in an anatomically realistic model of the upper cervical spine for a healthy subject (adult) and patient with CM (child). We chose these two model cases to understand how NRDL may impact CSF dynamics under a variety of flow conditions. Note that the intent of this study was not to find statistical differences between the patient and healthy case, but rather analyze the impact of NRDL. Our method was to perform CFD simulations on the healthy and CM models with and without NRDL included and quantify the impact of the presence of NRDL on CSF dynamics in terms of geometric, velocimetric and pressure-based parameters. The NRDL were idealized based on *ex vivo* cadaveric measurements in the literature. Herein, we describe the important findings and clinical applications of the CSF dynamics alterations that were observed due to the presence of NRDL.

### Geometric impact of the NRDL

An important finding of this work is that NRDL likely do not need to be included in CFD models of the upper cervical spine above C1. The presence of NRDL above C1 had little impact on the geometric parameters analyzed in this region (Table 1). This is due to the relatively small size that the NRDL have in relation to the overall SSS cross-sectional area near the FM. In the CM patient, the SSS was constricted at the FM and thus the NRDL had a greater impact on geometry, but even in this case the impact was small. The 1.5T MRI sequences did not have sufficient resolution to accurately detect NRDL however, there are new techniques such as 7T MRI [46] that may be able to capture NRDL. The present results support that even if MRI measurements obtained in the clinic had a greater resolution to quantify the NRDL, the geometric results would have little change rostral to C1. However, it should be noted that depending on the degree of tonsillar herniation, the SSS can be nearly obliterated and thus a higher resolution MRI measurement may still have importance to accurately quantify SSS in disease states such as CM. The CM patient for this study had relatively mild tonsillar herniation past the FM (5.8 mm).

While the NRDL did not alter craniovertebral geometry to a great degree, NRDL had a great geometric impact elsewhere in the cervical spine. This was due to the narrowing of the SSS along the cervical spine while the NRDL remained similar in size; thus the relative contribution of the NRDL increased moving caudally. On average, inclusion of NRDL made 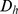 and 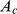 in the patient decrease by ∼30 and 5%, respectively and in the healthy subject decrease by ∼40 and 10%, respectively (Table 3 and Table 4).

The measured values for hydraulic diameters and cross-sectional areas in this study are within the range of previously published studies [1], [21]. Stockman was the first to include fine structures in a computational simulation of the SSS [26]. In this study, the SSS was idealized as an annulus with an elliptical cross-section and idealized NRDL's were added to the geometry symmetrically. The NRDL and arachnoid trabeculae were modeled as circular rods placed radially around the spinal cord with the spinal cord located concentrically within the dura both having a constant diameter along the SSS (2D symmetry). However information was not provided on the exact size of the NRDL. The arachnoid trabeculae were modeled to be 137 µm in diameter, a value one order of magnitude larger than that reported in the literature around the optic nerve of ∼25 µm [47]. This study found that the NRDL and arachnoid trabeculae had an impact on transport of substances such as drugs through the SSS. CSF velocity profiles were not altered to a great degree by the presence of NRDL and/or arachnoid trabeculae. This is likely due to the symmetry within the model geometry. The present study and *in vivo* anatomy do not have symmetry. Thus velocity profiles were skewed.

### NRDL result in elevated and inhomogeneous CSF dynamics

Our simulation results including the NRDL resulted in a number of important CSF flow features many of which have been observed by *in vivo* MRI measurements but not previously reported in CFD simulations. These CSF flow features include 1) elevated peak CSF velocities due to NRDL, 2) anterior and anterolateral dominance of CSF flow, 3) CSF flow jets between dorsal and ventral nerve roots and denticulate ligaments, 4) increased mixing of CSF (also described by Stockman[26]) and formation of vortical structures due to the NRDL throughout the cervical spine, and 5) elevated WSS with the NRDL.

For the first time in the literature, our simulations show that peak CSF velocities increase with the addition of NRDL. The impact of NRDL is most evident in the lower cervical spine (Table 3 and 4 and Figure 4) likely due to the narrowing of the SSS cross-sectional area moving caudally. As expected, the increase in CSF velocities corresponds with a decrease in the cross-sectional area of the SSS due to the NRDL. The magnitude of peak systolic and peak diastolic velocities in our simulations are similar to previous simulations [20], [24], [48]. For the CM patient, elevated CSF velocities were observed in the posterior SSS compared to the anterior SSS at the FM level (Figure 5); a result similar to that simulated by Linge et al. and Roldan et al. [22], [49]. However, the peak CSF velocities in these simulations are generally smaller in magnitude over the entire volume of space analyzed when compared to *in vivo* measurements [14], [24], [50].

Assessment of Reynolds number for external and internal flow indicated laminar flow in all CFD simulations. As expected, average Reynolds number for internal flow was greater in the CM patient than healthy subject (270 compared to 141). This is due to the smaller cross-sectional area for the CM patient in comparison to the healthy subject. Interestingly, average Reynolds number for external flow showed the opposite trend, the healthy subject was 215 and the patient was 168.

CSF velocity profiles with and without NRDL showed large differences in all regions of the cervical spine except at the FM (Figure 5 and Figure 6). Due to the low Reynolds number, any flow perturbations caused by the NRDL lower down in the SSS did not propagate upwards to the FM. The NRDL resulted in anterior and anterolateral dominance of CSF flow at most levels of the spine (Figure 5). While this pattern was present in both the healthy and patient case, it was more evident in the healthy case; likely due to the relatively larger size of NRDL and their tighter placement in the SSS. Anterior and anterolateral dominance of CSF flow was reported in previous *in vivo* PC-MRI measurements of CSF velocity in SSS [1], [14], [15], [24], [50]. This flow feature was not seen in the CFD simulation of CSF in the cervical spine without NRDL of Yiallourou et al. [24] and thus the NRDL included in our simulation likely play a role in this discrepancy.

The presence of NRDL was found to result in CSF jets between the dorsal and ventral nerve roots and denticulate ligaments (see C3 through C7 in Figure 5 and also Figure 6). These jets were not present in the upper cervical spine where the NRDL were not present and were greatest in the healthy case at the levels C3-C7. None of the previous simulations of CSF flow in the cervical spine has quantified any such flow jets between NRDL because these simulations did not include these small anatomical structures.

Bidirectional velocity, with a minimum magnitude of 1.6 cm/s, was observed in our simulation at the reversal of CSF flow in both healthy and patient cases over a duration of 0.21 s and 0.11 s, respectively. Thus, the magnitude and duration of bidirectional velocity increased in the presence of NDRL, suggesting that these structures may have a significant role in increasing the resistance against CSF flow in cervical SSS. The existence of synchronous bidirectional CSF flow was reported for both healthy and patient cases in previous CFD studies of spinal CSF flow [20], [48], [49] however the threshold criteria was not reported in these studies. It has also been noted in symptomatic CM patients using *in vivo* flow measurements [1], [15], [50], [51]. This flow feature was suggested to be an important clinical parameter indicating the increased resistance to CSF flow due to partial obstruction of the SSS [48].

The increased and prolonged bidirectional velocity, due to the presence of NRDL, was accompanied by the formation of vortical structures (Figure 7), similar to that observed *in vivo* by 4D PC MRI in the SSS [1]. However, the vortical structures observed by 4D PC MRI *in vivo* were only detected near the FM in CM patients. Our simulation results show that the vortical structures were present throughout the entire cervical spine in the healthy and CM patient model with NRDL. Also, velocity fluctuations of 1.01 and 2.15 Hz were quantified downstream of the NRDL for the healthy subject and CM patient, respectively. The lack of vortical structures in the *in vivo* 4D PC MRI measurements could be a result of the high velocity encoding value used for the 4D PC MRI measurements that did not capture vortical structures with lower CSF velocities and spatial resolution of the MRI. Inclusion of NRDL can be important in studying of mixing characteristics of CSF flow in SSS, as the formation of vortical structures during the flow reversal was neither observed in models without NRDL nor reported in previous CFD studies based on simplified geometries of SSS [20], [21], [24], [49].

Further insight on the transport behavior of CSF flow in the presence of NRDL can be obtained by comparing the magnitude of non-streamwise components of velocity vectors in the SSS over the cardiac cycle or SFP parameter (Figure 8). Elevated SFP in the presence of NRDL was evident to the greatest degree during CSF flow reversal. This is consistent with the formation of vortical structures near the NRDL during the same time interval (see streamlines in Figure 7) and suggests that the duration of bidirectional velocity can be regarded as a parameter directly influencing the mixing characteristics of the CSF flow field.

Mixing of CSF due to the presence of NRDL throughout the SSS may influence transport behavior of CSF chemicals and CNS drugs. Coupling a drug advection-diffusion model to the present model can assess the impact of NRDL on dispersion and diffusion. Hsu et al. constructed a subject specific model of the central nervous system to understand dynamics of intrathecal bolus injection [52]. They found that the speed of drug transport was strongly affected by the frequency and magnitude of CSF pulsations. Stockman [26] reported that fine structures can significantly increase non-streamwise components of CSF velocity and this may have a large effect on mixing and chemical transport behavior inside SSS. Further study into the importance of anatomy and CSF dynamics in the SSS could be helpful for improvement of intrathecal drug delivery devices and methods.

NRDL were found to increase WSS throughout the SSS, with the greatest WSS values occurring between the dorsal and ventral nerve roots. This was a result of the flow jets that occurred in these regions (Figure 5 and 6). The values of peak WSS in this study fall within the range reported by Loth et al. [21] (0–1 Pa). It is not known if these levels of WSS would have any impact on CNS tissue such as the pia and arachnoid mater. These delicate tissues could be altered under small stresses. Recent work has investigated the possible importance of mechanotrasduction of WSS due to CSF movement over the ependymal cilia [53], [54]. WSS due to blood flow has been examined extensively and shown to have a role in vascular endothelial function and pathological conditions [55]. The assessment of WSS caused by CSF flow in the SSS may also prove to have similar significance and could be investigated in future work.

### Impact of NRDL on pressure based parameters

Pressure gradients are the driving force responsible for CSF flow and neural tissue motion. Abnormal pressures and their resulting forces may alter the mechanical properties of neural tissues over time and can be the cause of neurological symptoms related with different craniospinal disorders [56]–[59]. Thus, a detailed understanding of the pressure environment in the CSF system is important. However, information obtained from MRI techniques are limited to geometry and velocity measurements and absolute pressure evaluation is not possible. *In vivo* invasive pressure recordings are possible, however their practical difficulties and inherent inaccuracy due to the alternation that is introduced in pressure environment, make their applications limited. CFD, on the other hand provides a non-invasive tool for calculating pressure gradients as well as velocity in SSS. However, the mean pressure is not possible to compute.

Our results support that inclusion of NRDL in CFD simulation of the upper cervical spine is not needed when pressure gradients within the FM-C1 region are of interest and tonsillar herniation past the FM is mild (∼6 mm). The peak pressure gradient within the FM-C1 region was nearly identical for the models with and without NRDL in both healthy and CM patient cases (see Table 3 and 4 FM-C1). This was due to the fact that NRDL occupied a relatively small amount of the SSS within this region. Thus, the results of previous studies in the literature focusing on the FM-C1 SSS region would likely not be altered if the NRDL were included provided that the simulation was performed for subjects with mild tonsillar herniation. However, in CM patients with a great degree of SSS abnormality, the NRDL could be abnormally positioned and thus alter pressure gradients within the FM-C1 region to a greater degree than in our limited simulation results. Interestingly, syringomyelia associated with CM rarely affects the C1 spinal cord.

While the CSF velocity field was altered to a great degree by the presence of NRDL, unsteady pressure gradient along the cervical spine had less than 20% increase (Figure 9). The addition of NRDL increased peak pressure gradient by 8 and 3 Pa for the healthy and patient cases, respectively. As expected, the healthy subject had a smaller peak pressure gradient (∼0.26 mmHg, average with and without NRDL) compared to the CM patient (∼0.32 mmHg). The greater pressure gradient in the patient was likely due to a smaller hydraulic diameter in the CM patient (Table 3 and 4). Peak pressure gradient computed for each of the eight axial sections of the model showed the greatest differences between the healthy subject and patient near the FM. This was also expected due to the smaller hydraulic diameter near the FM in the patient compared to the healthy subject. The pressure gradient results (Figure 9 and Tables 3 and 4) of this study are similar in magnitude to previously published CFD studies in the literature [20], [48], [60]. Linge et al. [20] Bilston et al. [60] and Cheng et al. [48] found a max pressure gradient of ∼0.45, 0.42 and 0.90 mmHg in the cervical region, respectively. In a fluid structure interaction model of the complete CSF system, Sweetman et al. found a maximum pressure gradient between the lateral ventricles and lumbar region of the SSS to be 1.04 mmHg [25].

ILI is defined in a way to quantify the viscous resistance in an unsteady flow field [61]. This parameter has been investigated to quantify the performance of arterial functions in normal and pathological conditions [62] and patency of vein grafts [43]. Similarly, the assessment of ILI, as a single parameter taking into account the impact of full three-dimensional geometry, may prove to be useful to quantify CSF flow blockage inside SSS and can be considered as a tool for diagnosing pathological conditions where CSF dynamics are thought to be an important factor.

Our results showed that partial obstruction of SSS with NRDL increased the magnitude of ILI as calculated through different segments and the whole cervical region. Similar to pressure gradients, the increase in ILI was small (Table 3 and 4). Specifically, similar to the trend observed for the variations of pressure gradient, it is seen that the impact of NRDL on ILI is negligible within the upper regions of cervical spine, mainly because of the absence or relatively small dimensions of the mentioned fine structures in these regions. Thus, the presence of NRDL can likely be omitted in CSF CFD simulations focusing on the FM region such as in CM patients with mild herniation.

### Limitations

This study included one CM patient and healthy volunteer as a platform to analyze the impact of NRDL on the CSF flow field. For this purpose, a larger study cohort would not be useful. However, to interpret the importance of NRDL under varying degrees of tonsillar herniation a larger cohort would be needed. To have meaningful results, such a study would require upper cervical spine models with subject-specific NRDL. This was not possible in the current study, as current MRI resolution limits do not enable quantification of NRDL.

To understand the impact of NRDL under a variety of CSF flow conditions, the healthy subject geometry used in this study was an adult while the patient was a child. Both geometries were obtained from supine MRI measurements. Shifting of the brain and/or spinal cord position due to posture changes was not analyzed. Since the anatomical data used in the modeling process of NRDL were obtained based on an adult population, NRDL were scaled in size (see methods). This study did not take into account the impact of other fine structures within the SSS, such as arachnoid trabeculae and blood vessels. It is yet unclear exactly what role these structures may play in CSF dynamics.

The CFD simulations conducted in this study were rigid walled and did not take into account motion of the neural tissues such as the tonsils and/or spinal cord that may be present in CM patients and healthy subjects in varying degrees [24], [63], [64]. A moving boundary or fluid structure interaction model of the upper cervical spine might lead to a more accurate CFD simulation.

## Conclusions

The presence of NRDL within the upper cervical spine was found to have an important impact on CSF dynamics in terms of velocity field and flow patterns. However, NRDL did not alter the flow dynamics to a great degree near the foramen magnum and rostral to C1 for a healthy subject and CM patient with mild tonsillar herniation (∼6 mm). In addition, the presence of NRDL did not change pressure distribution greatly. Overall, the NRDL increased fluid mixing phenomena and resulted in a more complex flow field. Arachnoid trabeculae and other small anatomical structures within the SSS should be analyzed in addition to NRDL. Also, the importance of tissue motion on CSF dynamics should be investigated in future studies.
